# Murine Models of Acute Myeloid Leukaemia

**DOI:** 10.3390/ijms20020453

**Published:** 2019-01-21

**Authors:** Marwa Almosailleakh, Juerg Schwaller

**Affiliations:** Department of Biomedicine, University Children’s Hospital beider Basel (UKBB), University of Basel, 4031 Basel, Switzerland; m.almosailleakh@unibas.ch

**Keywords:** acute myeloid leukaemia, AML, mouse models, transgenic mice, bone marrow reconstitution, genome editing, patient-derived xenografts, PDX

## Abstract

Acute myeloid leukaemia (AML) is a rare but severe form of human cancer that results from a limited number of functionally cooperating genetic abnormalities leading to uncontrolled proliferation and impaired differentiation of hematopoietic stem and progenitor cells. Before the identification of genetic driver lesions, chemically, irradiation or viral infection-induced mouse leukaemia models provided platforms to test novel chemotherapeutics. Later, transgenic mouse models were established to test the in vivo transforming potential of newly cloned fusion genes and genetic aberrations detected in patients’ genomes. Hereby researchers constitutively or conditionally expressed the respective gene in the germline of the mouse or reconstituted the hematopoietic system of lethally irradiated mice with bone marrow virally expressing the mutation of interest. More recently, immune deficient mice have been explored to study patient-derived human AML cells in vivo. Unfortunately, although complementary to each other, none of the currently available strategies faithfully model the initiation and progression of the human disease. Nevertheless, fast advances in the fields of next generation sequencing, molecular technology and bioengineering are continuously contributing to the generation of better mouse models. Here we review the most important AML mouse models of each category, briefly describe their advantages and limitations and show how they have contributed to our understanding of the biology and to the development of novel therapies.

## 1. Introduction

Acute myeloid leukaemia (AML) is a disease of an uncontrolled clonal proliferation of abnormal myeloid stem and progenitor cells in the hematopoietic tissue. The transformed myeloid cells or ‘leukemic blasts’ exhibit aberrant differentiation and accumulate in the bone marrow (BM). This process diminishes normal haematopoiesis, often leading to thrombocytopenia and anaemia, hematopoietic failure and mortality [1]. The genomic landscape of AML has been extensively studied since the 1970s, starting by the examination of chromosomal karyotypes of patients’ leukemic cells [1,2]. Several prevalent balanced chromosomal rearrangements, including t(8;21)(q21;q22), inv(16)(p13q22) and t(15;17)(q22;q12) were identified in tumour cells from AML patients and molecularly characterized [3,4]. All three of these rearrangements share the remarkable feature of generating chimeric fusion proteins, in which at least one of the fusion partners is a gene encoding for a transcriptional regulator that is required for normal haematopoiesis. The advent of higher resolution next generation sequencing (NGS) has led to the identification of additional recurring and singleton alterations including cytogenetically-silent translocations, point mutations in metabolic regulators and small copy number changes [5]. NGS studies also revealed that despite the detection of recurrent genomic aberration, the majority of the genomes of de novo diagnosed AML contain fewer number of mutations compared to most solid tumours [6]. A comprehensive NGS landmark study by Papaemmanuil and colleagues has identified 5234 driver mutations in 76 genes from a cohort of 1540 AML patients [1]. Recurrent AML-associated mutations can be grouped into different categories according to their functional consequences: those which are involved in epigenetic regulation such as DNA methylation (e.g., *DNMT3A, TET1, TET2, IDH1, IDH2*) and chromatin modification (e.g., *EZH2, ASXL1, KMT2A/MLL*), cellular signalling pathways of proliferation and survival (e.g., *FLT3, N-RAS, K-RAS*), key transcriptional regulators of haematopoiesis (e.g., *CEBPA, RUNX1, GATA2*), tumour suppressor genes (e.g., *TP53, WT1, PHF6*), RNA splicing (e.g., *SRSF2*, *U2AF1*, *SF3B1*) and formation of cohesions complex and chromatin architecture (e.g., *SMC1A, SMC3, STAG2*) [1,7]. In fact, recent functional studies revealed that in significant number of patients without a detectable cytogenetic aberration, AML emerges from functional cooperation of multiple alterations (e.g., *DNMT3A, TET2, IDH*, spliceosome mutations) that are often identified as molecular markers of potential pre-leukemic states such as clonal haematopoiesis of indeterminate potential (CHIP) and myelodysplastic syndromes (MDS) [7].

**Figure 1:**
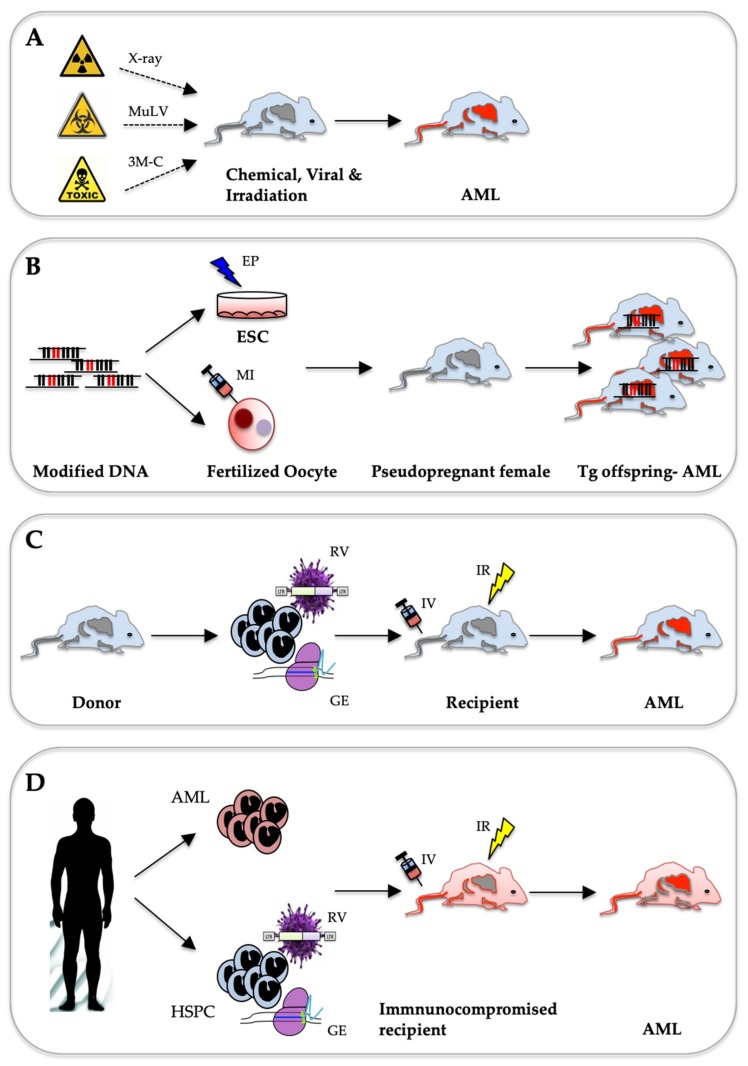
Schematics of different strategies for remodelling AML in mice. (**A**) Spontaneous AML development upon exposure to carcinogens like chemicals (e.g., 3-methylcholantrene; 3M-C), biologicals (e.g., murine leukaemia virus, *MuLV*) or radiation (X-rays). (**B**) Conventional transgenic approach: Transgenic (Tg) mouse lines are generated by DNA insertion into the genome, either randomly by pronuclear microinjections (MI) into fertilized Oocytes, or targeted by electroporation (EP) and homologous recombination in embryonic stem cells (ESC). (**C**) Adaptive transfer method of in vitro modified murine HSPC cells using either retroviral transduction (RV) or genome editing (GE) techniques followed by tail intravenous (IV) transplantation in irradiated (IR) recipients. (**D**) Xenotransplantation of either leukemic blasts or in vitro modified HSPC into immuno-compromised mice intravenously (IV) injected into irradiated (IR) recipients.

Although improved modern technologies have simplified the detection of genetic alterations in AML cells, the challenge remain in validating their function during initiation and development of the disease. These alterations are categorized as either potential driver mutations necessary for disease induction and/or maintenance or neutral passenger mutations that may not be part of disease aetiology. Despite improvement in ex vivo cell culture systems, significantly expanding primary AML blasts while preserving their naïve characters over a long period remains a challenging task [8]. In addition, significant patient-to-patient cell heterogeneity complicates studying common mechanisms that control AML biology. Thus, comprehensive functional characterization of many pathogenic phenomena could only be addressed using in vivo animal models, in particular, in genetically modified mouse strains. Unfortunately, despite collective efforts from many laboratories around the world, none of the existing models ideally recapitulate all aspects of the human disease. Nevertheless, the fast development of molecular and genetic engineering approaches has led to considerable progress. Faithfully modelling the complex heterogeneity of human AML in vivo will ultimately result in a better understanding of the molecular pathogenesis of the disease, identify genetic markers with predictive and prognostic value and develop novel personalized and efficient treatments strategies. Currently, mouse leukaemia models range from carcinogen-induced tumours, to transgenic animals expressing AML-associated proto-oncogenes and xenograft models based on transplantation of primary patient cells into immune-compromised mice (Figure 1).

## 2. AML Mouse Models Induced by Chemicals, Viral Infection or Irradiation

A large number of studies that date from the last century have shown that AML can be spontaneously triggered in mice by chemical compounds, irradiation or particular viral infections (Figure 1A). Notably, modelling chemically and irradiation-induced AML also accounts for the effects of the environment, which is mostly disregarded in genetically engineered transgenic or xenograft leukaemia models.

### 2.1. Chemically Induced Leukaemia Models

One of the first reported and widely used leukaemia model is the L1210 cell line (L stands for Lloyd Law) isolated from DBA/2 mice exposed to the carcinogen 3-methylcholantrene [9,10]. The cells can be propagated in vitro and give rise to secondary leukaemia when transplanted. This model permitted the study of disease initiation, kinetics and effectiveness of newly developed leukaemia therapies [11]. The majority of chemotherapeutic agents, such as the widely used cytarabine, were selected for AML therapy during the late 1960s based on the in vivo efficacy against leukemic L1210 and other similar leukaemia models (P388, P1534 and L5178Y) [12]. However, the use of such cell line-based models had significantly diminished in the last decades due to several limitations. First, the pathology of the leukaemia induced by these cells does not fully phenocopy human AML, as mice often develop a lymphoid disease. Secondly, only a small number of animals develop the disease after a long latency on exposure to the inducing carcinogen [13]. Therefore, the study of AML development in individuals as a consequence of chemical exposure (e.g., benzene) [14,15,16] or as results of chemotherapy (e.g., alkylating agents and topoisomerase II inhibitors) [17,18] have relied mainly on epidemiological and direct analysis of primary patient-derived material, rather than on the use of mouse models [19].

### 2.2. Radiation-Induced Leukaemia Models

Leukaemia was one of the first malignancies reported as a radiation-induced cancer. Leukaemia incidents were significantly higher among X-ray workers and scientists working in close proximity to particle accelerators, especially before the introduction of safety measures [20]. Survivors of the atomic bomb explosion in Hiroshima and Nagasaki were exposed to high doses of irradiation of high energy radiation which resulted in rather rapid increased risk for developing haematological malignancies in particular chronic myeloid leukemia (CML), acute lymphoblastic leukemia (ALL) and AML [21]. The Chernobyl accident on the other hand, resulted in exposure to isotopes of lower energy which significantly increased the risk to develop thyroid cancers but was associated with a much lower risk of developing hematologic malignancies [22]. Multiple murine strains develop leukaemia on exposure to high and low-grade radiation, including the RFM, CBA, C3H and SJL/J [23]. Single high dose or prolonged low-grade full body irradiation such as gamma radiation, X-rays and neutrons has reported to induce leukaemia or mixed leukaemia/lymphoma development in mice.

Radiation-induced AML (RI-AML) in the RFM mouse line correlates with human data with comparable time of exposure to leukaemia development latency [24,25]. The clinical presentation of RI-leukaemia in the SJL/J mouse closely resembles that of secondary human AML, occurring after radiation therapy of patients with Hodgkin’s disease [26]. One of the most interesting findings was that the incidence of RI-AML in SJL/J mice increased upon co-administration of corticosteroids and colony-stimulating factor-1 (CSF-1). This correlated with human findings where higher expression of CSF-1 could be associated with poor outcome in AML [27,28]. Strikingly, the most common cytogenetic feature detected in RI-AML models was recurrent deletions of chromosome 2. The identification that the minimal deleted region contained the *Sfpi1* gene encoding for the PU.1 transcriptional master regulator of myeloid differentiation shed some light on the underlying mechanism of disease initiation [25]. Later studies have shown that loss of one *Sfpi1* (PU.1) allele is not sufficient to induce a myeloid malignancy, despite the cells having a growth advantage [29]. A “second-hit” in these cells, in the form of a point mutation in the second *Sfpi1* (PU.1) allele in its DNA binding domain (R235), is believed to transform these cells leading to clonal expansion and cancer [30,31]. Sequencing of AML samples from survivors of the Chernobyl accident showed similar mutational pattern with large chromosomal deletions and loss-of-heterozygosity (LOH) in multiple locations in the genome [32]. Experimental irradiation was also shown to accelerate the development of leukaemia in engineered mouse models, for example, such as the acute lymphoblastic leukaemia (ALL) associated with t(12;21)(p13;q22) leading to a *TEL-AML1* (aka *ETV6-RUNX1*) fusion, coupled with a loss of the *CDKN2A* cell cycle regulator gene [33]. This finding supported a model in which environmental low-grade radiation exposure may induce cooperating mutations to existing initiation lesions resulting in the expansion of pre-leukemic clones. Understanding the underlying molecular pathogenic mechanisms leading to RI-AML would help for radiation mitigation and to develop better radio-protective agents to reduce the incidence of secondary malignancies.

### 2.3. Virally Induced Leukaemia Models

Murine leukaemia viruses (*MuLV*) have been widely used to model the disease in susceptible mouse strains [34]. Pioneering work in the 1950s demonstrated that leukaemia could be induced and serially transmitted by injecting cell-free filterable *MuLV* supernatants into new-born mice [35]. Historically, murine leukaemia viruses were named after the scientist who originally characterized them, such as *Gross-MuLV*, *Friend-MuLV*, *Moloney-MuLV*, *Graffi-MuLV* and *Rauscher-MuLV*. Each of these virus strains results in recognizable and predictable patterns of disease in particularly susceptible mice strains, such as NIH/Swiss, DBA/2, AKXD, BXH-2 and C57BL-6 [36]. The original Friend virus preparation contains two retroviruses, a defective spleen focus forming virus (*SFFV*) and a replication competent murine leukaemia virus (*F-MuLV*) [37,38]. Two different *SFFV* strains have been identified: (*SFFV_P_*) which reproducibly lead to polycythaemia and (*SFFV_A_*) which results in anaemia. The target cell in which both *SFFV* express their pathogenic effect is an erythropoietin (EPO)-responsive progenitor cell identified as a late erythroid burst forming unit (BFU-E) or colony-forming unit (CFU-E). The envelope protein encoded by *SFFV* interacts with and activates the EPO receptor and sf-Stk (a truncated form of the Stk/RON receptor tyrosine kinase) causing EPO-independent proliferation, differentiation and survival. In the second stage, *F-MuLV* integration into the *Sfpi1* locus activates the myeloid transcription factor PU.1, blocking erythroid cell differentiation. Cells from diseased mice can be serially transplanted in vivo and propagated as permanent cell lines in vitro known as murine erythroleukemia (MEL) cells [39]. Subsequent studies suggested that aberrant PU.1 expression leading to functional inhibition of the GATA1 major erythroid transcriptional regulator is the causal event for blocked terminal differentiation [40,41].

Virally induced AML was also studied in the AKXD (recombinant inbred strain derived from AKR/J expressing two endogenous MuLV, *Akv-1* and *Akv-2*, and DBA/2J.) inbred mouse strain to identify putative leukaemia-inducing oncogenes through insertional mutagenesis [42]. Notably, the *ecotropic virus integration site-1* (*EVI1*) gene on 3q26 in the human genome, today a well characterized molecular marker in aggressive AML, was identified by analysis of retroviral integration site in *MuLV*-infected diseased AKXD mice [42]. Although a conclusive link between viral infection and AML induction in humans was never established, the use of these models has been instrumental for the identification and function of many AML-associated proto-oncogenes and the development of anti-leukemic therapeutic strategies. Improved molecular tools such as NGS for genome-wide viral integration site definition and the subsequent development of more sophisticated viral strains [43] resulted in several high-throughput insertional mutagenesis screens using both virus- or transposon-based (e.g., *Sleeping Beauty*) systems [44,45,46]. This approach of forward genetics was critical in identifying many cooperating proto-oncogenes that accelerate leukaemia development and eventually confer drug resistance [47].

## 3. Genetically Engineered Mouse Models

The molecular revolution in biological methods in the 70–80s of the last century allowed researchers to transfer foreign genetic elements into the germline of mice to create homogenous transgenic lines. To study the activity of putative proto-oncogenes, researchers integrated expression cassettes and mini-genes with promoter/enhancer elements, open reading frames (ORF) and transcript stabilization elements. A linearized copy of the engineered DNA is introduced by either direct injection into mouse oocytes or by electroporation into mouse embryonic stem (ES) cells (Figure 1B). Driven by the success of modelling B-cell leukaemia/lymphoma in transgenic mice expressing the *c-myc* oncogene under the control of immunoglobulin heavy chain (*IgH*) gene promoter [48,49], several conventional transgenic AML mouse lines were generated (Table 1). Further refinement of the technology rapidly increased the number of AML models driven by proto-oncogenes controlled either by their endogenous promoter or by inducible expression from a heterologous promoter.

### 3.1. Conventional Transgenic AML Models

The “classical” approach to establish a transgenic AML mouse model is based on direct injection of DNA fragments containing an ORF of the desired genetic and regulatory sequences into the pro-nucleus of fertilized oocytes. The zygotes are then transplanted into pseudo-pregnant foster mother mice. This results in a random integration of the transgene and founder animals are typically identified by either restriction enzyme digests and Southern blotting or genomic PCR assays [50]. Several groups explored this strategy to establish transgenic models for the *PML-RARA* fusion gene resulting from the t(15;17)(q24;q21) chromosomal translocation present in the vast majority of patients with acute promyelocytic leukaemia (APL). Hereby different regulatory elements directing transgene expression towards the myeloid lineage derived from human/mouse *cathepsin G (CG)* [51], *CD11b* [52] or *MRP8* [53] (*S100A9*) genes were used. The rather wide spectrum of the resulting phenotypes illustrates the complexity and limitations of this classical transgenic approach. Whereas expression of *hCG* or *MRP8* controlled *PML-RARA* expression was able to induce AML or APL-like phenotypes with incomplete penetrance after long latency [51,53], *CD11b/PML-RARA* transgenic mice did not develop any leukaemia [52]. Nevertheless, classical transgenic mice were instrumental to show that *PML-RARA* indeed is the genetic driver of APL and to study the underlying molecular mechanisms leading to the first (and so far only) really efficient targeted AML therapy based PML-RARA degradation by all-trans-retinoic acid (ATRA) and/or arsenic trioxide [54]. Notably, another classical transgenic model for APL associated with a *PLZF-RARA* fusion gene derived from t(11;17)(q23;q21) revealed that ATRA was unable to induce remission in mice, faithfully recapitulating the clinical response in the respective patients [55]. We list some of the most important classical transgenic AML models in Table 1 and refer to respective review articles [56,57,58]. Unfortunately, the classical transgenic mouse approach is inefficient, technically challenging, time- and cost-consuming and as illustrated by the APL mouse model and others, unable to recapitulate the desired phenotypes [59]. Therefore, it is most likely that a large number of classical transgenic mouse lines expressing leukaemia-associated proto-oncogenes that were not able to phenocopy human disease remained unpublished.

### 3.2. Transgenic AML Models by Homologous Recombination in ES Cells

The site of integration of the cloned DNA can be specifically directed toward the desired gene locus thanks to the development of targeted homologous recombination (HR) in murine embryonic stem (ES) cells [67]. This technology was explored early on to establish AML mouse models and a positive proof of concept was provided by the first transgenic model for the *MLL-AF9* fusion gene associated with myelomonocytic leukaemia [68]. Physiologically, the mixed lineage leukaemia (*MLL*; aka *KMT2A*) gene encodes for a regulator of self-renewal and differentiation of hematopoietic stem cells (HSC) and is target of recurrent chromosomal translocations that lead to fusions of its amino-terminus to the carboxy-terminus of one of > 60 different partner loci [68]. MLL fusion genes are the molecular hallmark of more than 70% of infant acute leukaemia, 5–10% of adult de novo AML and an increasing number of secondary and therapy-related AML [69]. The most prevalent translocations comprise t(9;11)(p22;q23), t(11;19)(q23;p13) and t(4;11)(q21,q23) leading to the *MLL-AF9*, *MLL-ENL* and *MLL-AF4* fusion genes, respectively [69]. To express the *MLL-AF9* fusion from its native regulatory elements, Rabbitts and co-workers successfully integrated a short *MLL exon8-AF9* cDNA-poly-A fragment into the mouse *Mll1* locus by homologous recombination [70]. Interestingly, despite the widespread activity of the *Mll1* promoter, chimeric mice only developed AML. Notably, joining *Mll1* exon8 with a bacterial *lacZ* gene was sufficient to induce leukaemia in some chimeric mice after prolonged latency [71]. Subsequent studies with the *Mll-AF9* knock-in mouse line demonstrated pre- and postnatal stepwise progression of the disease [72], the role of the HOXA9 homeobox transcription factor as downstream effector [73] and gene dosage effects as well as putative cellular targets of MLL-AF9 to initiate AML [63].

The success of modelling AML by constitutive integration of a driver fusion oncogene into its natural locus encouraged researchers to model the function of other alterations. The fusion genes of the core-binding factor (CBF), a heterodimeric essential HSC regulator composed of RUNX1 (AML1) bound to CBFβ, is involved in balanced chromosomal rearrangements found in 20–30% of human AML [74]. RUNX1, was initially identified as a target of the t(8;21)(q21;q22) chromosomal translocation which results in expression of a fusion protein that contains the N terminus of RUNX1 fused to a nearly full-length ETO (Eight-Twenty-One, aka RUNXT1 or MTG8) protein. Knocking-in the *RUNX1-ETO* fusion gene into the murine *Runx1* promoter lead to embryonic lethality and a lack of definitive haematopoiesis in the foetal liver, very similar to those seen in *Runx1^−/−^* knockout mice [75]. CBF is also target of inv(16)(p13q22) leading to expression of a *CBFβ-MYH11* fusion gene. The resulting fusion protein was shown to interact with RUNX1 and to outcompete binding of wild-type CBFβ in a dominant-negative fashion. Not surprisingly, expression of a single copy of *CBFβ-MYH11* from the *Cbfb* promoter (*Cbfb^+/MYH11^*) resulted in a similar lethal phenotype as *Runx^−/−^* mice [76]. Thus, to be able to study the role of these CBF fusions for leukemogenesis in vivo, it was essential to express them in a spatially and temporally-controlled manner.

### 3.3. Conditional Transgenic AML Mouse Models

#### 3.3.1. Modelling AML-Associated Fusions

Conditional gain-of-function models of AML-driving (fusion)-oncogenes are mostly generated by inserting a strong translational and transcriptional termination (STOP) sequence flanked by *LoxP* or *Flp* recombinase recognition target site (FRT) cleavage sites between the promoter sequence and the ORF of interest. In presence of a C- (Cre) or FLP recombinase, the STOP cassette is removed, allowing the expression of the transgene. The same approach can also be used to ablate essential parts of a gene of interest by deleting regions flanked by *LoxP* sites. Utilizing this setting, transgenic mice carrying the floxed genes are usually crossed with transgenic lines that express the Cre recombinase in the hematopoietic tissue. Cre expression is typically driven under the control of spatially and/or temporally controllable promoters such as the hematopoietic-restricted *Vav1* (*Vav1-iCre*) promoter, the interferon-inducible *Mx1* promoter (*Mx1-iCre*) or as a fusion to a mutated oestrogen receptor (ER) ligand binding domain (*Cre-ER*), which can be activated by tamoxifen (TAM) [77].

An additional layer of complexity can be added by engineering transgenic cassettes controlled by minimal promoters that are sensitive to chemical inducers like tetracycline (Tet) [78], or one of its derivative such as doxycycline (DOX). The Tet system can be designed to inhibit (Tet-off) or induce (Tet-on) the expression of a transgene, by either coupling it with a Tet-sensitive transcriptional repressor (*tTA*) or a reverse-tTA (*rtTA*) transcriptional activator respectively [79]. A Tet-off expression system was applied to bypass the embryonic lethality associated with constitutive *RUNX1-ETO* expression [60]. Another study established Cre-responsive conditional *RUNX1-ETO* knock-in mice [80]. However, despite robust expression of the *RUNX1-ETO* fusion transgenes in the BM upon tetracycline withdrawal in the first model and efficient excision of the *floxed STOP* codon in the second model, no leukaemia developed. In subsequent studies, researchers showed that mice conditionally expressing *RUNX1-ETO* developed leukaemia only upon treatment with genotoxic agents such as N-ethyl-N-nitrosourea (ENU) [61]. ENU is a strong carcinogenic mutagen which transfers its ethyl group to oxygen or nitrogen radicals into DNA, resulting in miss-pairing and base pair substitutions which translates to the production of proteins with missense mutations and aberrant splicing events [81]. A *RUNX1-ETO* leukaemia model that allows for conditional and reversible controlled mosaic expression of the fusion in hematopoietic progenitors was established by transplanting whole BM carrying a *ROSA26-iM2-tetO* DOX inducible promoter and the fusion cDNA (*ROSA26-iM2-tetOGFP/TgPtet-AML1-ETO*) into lethally irradiated mice [82]. Hereby the researchers were able to recapitulate the slow disease evolution and mosaic expression found in human RUNX1-ETO^+^ AML. Transcriptional analysis from different hematopoietic populations during disease progression demonstrated that the fusion alters the transcriptional expression of HSC and committed progenitors. However, despite showing signs of a myeloproliferative leukaemia-like disease, all the mice survived. This finding is consistent with the idea that *RUNX1-ETO* expression is necessary but not sufficient to induce a fully penetrant AML. Indeed shRNA-targeted degradation of the fusion significantly reduced proliferation and survival of *RUNX1-ETO*-expression AML cells [83]. Later studies found functional cooperation of *RUNX1-ETO* with mutations in tyrosine kinases such as *c-KIT, FLT3-ITD* or the *TEL-PDGFβR* fusion in different mouse models [84,85].

A similar conditional mouse model was developed for the *CBFβ-MYH11* fusion gene, called *Cbfb^+/56M^* [62]. Wild-type *Cbfb* cDNA (exon 5 and 6 and a polyadenylation signal) flanked by *LoxP1* sites was inserted into intron 4 of the previously generated transgenic *Cbfb^+/MYH11^* knock-in allele. Hereby the wild-type *Cbfb* transcript is temporarily expressed from the ‘’floxed’’ *Cbfb^56M^* allele. However, in presence of Cre, the knock-in allele is restored and a *Cbfβ-MYH11* fusion is expressed. Strikingly following injection of polyinosinic:polycytidylic acid (poly(I:C)) activating *Mx1-iCre*, 90% of the mice developed AML after a median latency of 5 months demonstrating that the fusion is indeed a driver of AML [62].

We have established a series of Tet-regulated transgenic mice to model acute leukaemia driven by the most prevalent MLL fusion genes [65,66]. We were particularly interested to study the role of the cellular origin on AML onset and progression. Using this model, we were able to show that conditional expression of the MLL-AF9 fusion in long-term HSC (LT-HSC) resulted in a more aggressive phenotype than activation in the committed granulocyte-macrophage (GMP) or common myeloid progenitors (CMP) [65]. Notably, in a subset of mice, activation of MLL-AF9 led to a particularly invasive and drug-resistant phenotype characterized by expression of genes previously associated with epithelial-mesenchymal transformation (EMT) observed in solid cancers. Cross-species comparative gene expression profiling suggested that similar to MLL-AF9 driven AML in mice, some AML patients (not only those carrying MLL-fusions) expressed similar EMT-related genes associated with poor outcome [65]. In contrast to *MLL-AF9*, conditional expression of the *MLL-ENL* fusion using the same conditional Tet-on system was not able to transform GMP but induced a rather mixed myeloid-lymphoid leukaemia when activated in HSC, lymphoid-myeloid progenitor population (LMPP) or CMP [66]. Comparison with another Tet-regulated MLL-ENL transgenic mouse model suggested that the leukemic phenotypes might be influenced by the expression level of the transgene in cells of a particular stage of the hematopoietic hierarchy [64].

#### 3.3.2. Modelling AML-Associated Mutations and Aberrantly Expressed Genes

Several transgenic mouse models have been generated to model AML carrying NPM1 mutations [86]. Conventional transgenes in which expression of mutated *NPM1* was regulated by the human *MRP8* promoter [87] and a knock-in model mimicking the human mutation in the mouse *Npm1* [88] developed myeloproliferative disease only but no AML. Conditional ex vivo activation of a human *NPM1* mutant cDNA integrated in the *Hprt* locus followed by transplantation into irradiated WT mice induced a late-onset AML-like disease in about 30% of the recipients [89]. Conditional expression of a humanized *NPM1c* knock-in allele in the hematopoietic system (mediated by *Mx1-iCre*) resulted in the development of late onset AML in about 30% of the mice, however this percentage increased to 80% following the activation of cooperating proto-oncogenes through the use of the *Sleeping Beauty* insertional mutagenesis system [46]. Collectively, these models indicated that an NPM1 mutant is not sufficient to induce clinical AML.

Transgenic mouse models have also been established to model the role of the Flt3 (Fms-related tyrosine kinase 3) internal tandem repeats (FLT3-ITD) mutation found in > 20% of human AML [90]. Two independently established knock-in mouse lines carrying an ITD mutation in the juxta-membrane domain of murine *Flt3* slowly developed a myeloproliferative disease but no acute leukaemia [91,92]. However, it is important to note that *Flt3^ITD^* models were instrumental to demonstrate the impact of the gene dosage, loss of the wild-type allele and FLT3 ligand on phenotype development [93,94].

Very similar to FLT3 mutations, activation of conditional transgenic knock-in alleles of AML-associated *K-RAS^G12D^* and *N-RAS^G12D^* mutations resulted a highly penetrant myeloproliferative phenotype but was not sufficient to induce AML [95]. Thus, to be able to study cooperation between co-occurring mutations in AML, an increasing number of compound transgenic/knock-in mouse lines are generated (Table 2). For example, crossing the *NPM1c* with *Flt3^ITD^* knock-in strains revealed a powerful molecular synergy with the development of highly penetrant acute leukaemia [96]. Transgenic *Flt3^ITD^* expression was also shown to cooperate with *Mll^PTD^* [97], the *NUP98-HOXD13* fusion [98], the *Wt1^R394W^* [99] mutation or with *Dnmt3a* [100] haploinsufficiency to cause AML. Potent in vivo oncogenic cooperation was also demonstrated by crossing the *N-Ras^G12D^* knock-in strain with transgenics expression of the *MLL-AF9* fusion gene [101], the anti-apoptotic regulator *BCL2* [102] or the *Cbfβ-SMMHC* fusion [103]. Transgenic expression of *K-RAS^G12D^* increased the penetrance of the APL-like phenotype in cathepsin-G driven *PML-RARA* transgenic mice [104]. Collectively, mouse models have shown that AML-associated NPM1c, FLT3-ITD and N-/K-RAS mutations are *per se* not sufficient to induce the disease but act as potent cooperating lesions.

A transgenic mouse line remodelling the aberrant expression of the *EVI1* gene mediated by 3q21-3q26 chromosomal translocations or inversions leading a hallmark of particularly aggressive AML was recently established [105,106]. All the breakpoints detected in patients cluster within an approximately 25kb region, which in the mouse maps to -77 kb upstream of the *Gata2* gene. To test whether this region possess enhancer activity, researchers established a transgenic mouse line with a fluorescent reporter cloned 186 kb downstream of 5’ sequences flanking the *Gata2* gene [106]. Strong reporter signal was detected in HSPC and the sequence was thus designated as the *Gata2* distal hematopoietic enhancer (G2DHE). The same researchers then established a bacterial artificial chromosome (BAC) transgenic mouse that allowed the induction of *EVI1* expression with or without *G2DHE* region. All mice with an intact *G2DHE* developed leukaemia in accordance with transgene copy number, where two copies gave rise to B-cell, three copies resulted in myeloid and four copies led to mixed lineage leukaemia within 200 days. However, mice lacking the *G2DHE* region did not show sign of disease during the 400 days of observation indicating that the GATA2 enhancer plays a critical role [106]. This study confirmed and extended the observations that genomic excision of a distal *GATA2* enhancer led to *EVI1* silencing, growth inhibition and differentiation of human AML cells with inv(3)(q21q26) or t(3;3)(q21;q26) [107]. More recently, a transgenic mouse line was established in which *EVI1* expression is under the control of a Tet inducible (tet-on, “TO”) promoter (*Evi1^TO/+^/Rosa26^rtTA^*) [108]. To recapitulate the clinical presentation of EVI1 overexpressing leukaemia, researchers performed competitive 1:1 transplantation with *Evi1^TO/TO^*/*Rosa26^rtTA^* with WT BM cells. Using this approach all mice developed symptomatic AML within 90–119 days, clearly demonstrating its oncogenic activity [108].

Transgenic mouse models of leukaemia have been vital for our understanding of the role of genetic aberration in the induction and maintenance of the leukemic condition. However, one of the main shortcomings of these models is their inability to reliably reproduce the leukemic phenotype observed in patients carrying the genetic lesion. Several factors could attribute for that; such as the evolutionary difference between the human and mouse haematopoiesis systems, the effect of unaccounted genetic variability in the human genome (e.g., SNP) and generating transgenic mouse lines with the cDNA sequence only. This might subsequently lead to the potential loss of essential regulatory elements located in the intronic regions of mutated genes, causing an alteration in the dynamic expression of the genetic lesion in targeted cells and thus phenotypic differences. For example, the dynamic expression of the *Gata1* gene in erythroid cells versus HSC was shown to be depended on untranslated regulatory elements located at its 5’ region [109,110].

## 4. Mouse Models Based on Adaptive Transfer of Hematopoietic Cells Virally Expressing an AML-Associated Proto-Oncogene

Technologies developed during last two decades of the 20th century allowed to transfer the cloned leukaemia-associated genetic aberrations into hematopoietic cells to explore their transforming potential in vitro and in vivo. Production of replication-incompetent high titre retrovirus expressing a gene of interest was critical to develop the widely used adoptive transfer protocol to model the effects of leukaemia-associated genetic lesions in hematopoietic stem and progenitor cells (HSPC) of the mouse. Hereby, virally transduced cells are transplanted into lethally or sub-lethally irradiated syngeneic recipients, resulting in chimeric animals in which the donor-derived transformed HSPC may outcompete the host haematopoiesis ultimately leading to leukaemia (Figure 1C). The power of this strategy became first evident by studies of the Baltimore laboratory that modelled the effect of the chronic myeloid leukaemia (CML)-associated *BCR-ABL* fusion gene [111]. Transplantation of BM cells transduced with a retrovirus carrying the *BCR-ABL* fusion cDNA induced hematologic malignancies in about half of the recipients: either a CML-like myeloproliferative syndrome, acute lymphoblastic leukaemia (ALL) or tumours containing macrophage-like cells occurring after mean latencies of 9, 14 and 16.5 weeks respectively. Notably they were able to transfer the disease phenotype by transplanting tumour cells into irradiated secondary recipients.

Following this landmark study, this approach, often referred to as the transduction-transplantation model, was further refined and successfully used to model the transforming activity of a large number of AML-associated genetic alterations [112]. BM reconstitution with HSPC expressing a gene or mutation of interest was not only instrumental to demonstrate the transforming potential but also to validate functional cooperation of different mutation classes necessary to induce a leukemic phenotype, such as transcription factor fusion genes involving *CBF*, *RARA* or *NUP98* cooperating with *FLT3* or *N-/K-RAS* mutations [113]. Some of the most important models that were established by this approach are listed in Table 3. Many of these studies suggested that most AML-associated mutations are not sufficient to induce the disease. The versatility of the system allowed researchers to define many critical downstream effectors of AML driver mutations. In addition, such studies also suggested that the cellular origin might be an important nominator of transforming potential of AML-associated mutations. Transduction of enriched hematopoietic stem and distinct progenitor cells showed that in contrast to *BCR-ABL*, AML-associated *MOZ-TIF2* [114], *MLL-AF9* [115,116], *MLL-ENL* [117,118], *AML1-ETO* [119] and *MLL-GAS7* [120] fusion genes were able to transform committed progenitor cells. In addition, selective expression in different myeloid progenitor cell populations (CMP vs. GMP) revealed a differential transforming activity of the of the *meningioma 1* (*MN1*) gene, often overexpressed in aggressive AML [121].

In the majority of these studies, researchers used replication-deficient *murine stem cell virus* (*MSCV*)-based expression vectors, which allow efficient transduction and stable transgene expression in hematopoietic progenitor cells [122]. However, it is worth noting that viral integration events, potentially non-physiological expression level, batch to batch transduction and transplantation variability and the inherent transduction bias for early multi-potent HSPC may influence the disease phenotype. Nevertheless, in general the AML disease arising in these mice share a common histopathological and immunophenotypic features, best illustrated by the MLL-AF9 fusion. Independ whether the fusion is expressed retrovirally or as a knock-in transgene, the resultant disease is characterized by extensive infiltration of the BM and other organs by myeloid progenitors and monoblasts expressing high levels of Gr1, Mac1 and c-Kit surface markers [63,65,115,116]. The adaptive transfer model is still the prime experimental method to investigate the in vivo transforming potential of AML-associated genetic aberration. It provides a relatively rapid and robust methodology to explore the function of one or more AML-associated mutations or overexpressed genes in cells of the hematopoietic system.

## 5. Modelling AML by Transferring Patient-Derived Cells into Immune-Compromised Mice

Ex vivo maintenance and expansion of even the most clinically aggressive patient-derived leukemic blasts remains a technical challenge. Even very sophisticated culture systems cannot fully replace the complex interactions between leukaemia cells and the BM microenvironment. To overcome these limitations researchers explored transplantation of human primary AML cells into immune compromised mice (Figure 1D). Several immunodeficient mouse strains were developed for patient-derived cell xenotransplants (PDX) including *nude* (*nu*), severe combined immunodeficient (SCID), non-obese diabetic (NOD), NOD-SCID and NOD-SCID-IL2rγ^null^ (NSG) strains [123,124].

In one of the first PDX experiments, researchers transplanted primary AML cells into *nude* mice that are athymic due to a homozygous *nude* mutation (encoding for a forkhead box transcription factor (FoxN1), resulting in lack of functional T cells. However, due to an intact B cell and NK cell function, grafting of normal as well as leukemic cells remained poor and was often associated with formation of extramedullary granulocytic tumours [125]. Even in mice carrying triple homozygous mutations in *nude*, *beige* (affecting the lysosomal trafficking regulator; *Lyst*) and *Xid* (X-linked immunodeficiency gene, Bruton’s tyrosine kinase; *Btk*) xenografting of human AML cells remained inconsistent and unreliable [126]. The development of severe combine immunodeficient (SCID) mice was an important step forward for the development of humanized AML mouse models. SCID mice carry inactivating mutations in the *protein kinase DNA-activated catalytic polypeptide* (*Prkdc*) gene, which protein product is involved in DNA repair pathways. This leads to improper immunoglobulin *V-D-J* gene recombination, subsequently resulting in mice lacking functional mature T and B cells, however retaining NK function [125]. Although primary AML injected intraperitoneally or implanted under the kidney capsules showed improved engraftment rates, intravenous injection remained poor [127]. To overcome these limitations, researchers began to transfer the cells directly into the recipients’ BM by intrafemoral injection. To further improve engraftment rates, mouse models with more severe immunodeficiency were developed by combining the SCID background with the non-obese diabetic (NOD) strain. Combined non-obese diabetic NOD-SCID mice have no functional B or T cells and reduced NK cell and macrophage activity [128]. They showed superior engraftment rate compared to SCID mice even when injecting fewer primary AML cells [129]. Moreover, the morphologic, phenotypic and genetic characteristics of the expanded AML specimens seemed mostly preserved [129]. The ability to initiate the AML from few number of phenotypically stable cells allowed researchers to propose the existence of an AML-cell hierarchy with leukemic stem cells (or SCID-Leukaemia initiating cells; SL-ICs) enriched in the lineage marker-negative CD34^+^/CD38^−^ compartment [130,131]. Crossing of NOD-SCID mice with *IL2Rγ^−/−^* mice resulted in an even more immune compromised (NOD/LtSz-*scid* with *IL2γ_c_^null^*; NSG) strain [132]. Deletions in the interleukin-2 receptor gamma chain (*IL2Rγ*) led to an almost complete absence of the murine immune system and improved AML engraftment [133]. To further humanize the hematopoietic system, the NSG strain was crossed with knock-in mice expressing genes of three human cytokines (*hIL3*, *hGM-CSF* and *hSCF*) (NOD/LtSz-*scid IL2γ_c_^null^*–SGM3; or NSG-S) [134,135]. NSG-S mice showed significantly improved expansion of normal human myeloid cells and enhanced engraftment rates of primary patient AML cells [134,136]. NSG strains carrying null alleles for major histocompatibility complex class I and class II beta2-microglobulin (β2m) called NSG-β2m were developed to minimize reactivity of human immune cells against host tissue and thus specifically reduce graft versus host disease (GVHD) [137,138]. Notably, increased in engraftment rate for AML cell lines and primary paediatric patient samples in these mice without the need for irradiation was reported [139]. More recently, NSG^W/V^ and NSG^W41^ mouse strains were obtained by breeding NSG with strains carrying *c-kit* loss-of-function alleles (*Kit^WV/WV^* and/or *Kit^W41/W41^*) [140]. Loss of *c-Kit* impairs HSCs of the host and thereby creating empty BM niches leading to a competitive advantage for transplanted human HSPC. These strains supported engraftment of human CD34^+^ cord blood cells (CBCs) without prior host irradiation. They also showed greater engraftment and appropriate differentiation of human cells of the erythroid and megakaryocytic lineages [141]. In addition to the severity of immunodeficiency of the host, expression of human engraftment-enhancing cytokines and creating empty niches in the BM, the mutational status of the AML cells and the observation time seem also key determinants for successful expansion in PDX [142,143,144].

Many compounds that showed significant anticancer effects in vitro and in transgenic mouse models failed to show efficacy in clinical trials, most likely due to the unaccounted complexity of the mutational load of human AML and effect of the microenvironment [145]. To circumvent this limitation, the PDX model has been suggested as a good system to evaluate the efficacy of chemotherapeutic agents on human AML cells in vivo [146,147]. Combination therapy of cytarabine and doxorubicin on freshly transplanted human MLL-AF9^+^ leukaemia in NSG mice resulted in a reduction in residual disease burden [147]. Doxorubicin treatment had a profound effect on AML cells compared to mouse BM cells, in contrast to cytarabine which had a greater toxic effect on mouse BM cells. Transplanted primary samples showed variable sensitivity to chemotherapy, correlating with patients’ clinical outcome [146]. In another study, transplantation of Ara-C-resistant primary human AML cells into NSG mice revealed a role for mitochondria and elevated oxidative metabolism in leukemic cells’ chemo-resistance [148]. Thus, the PDX system seem to provide an experimental platform to test the efficacy of novel therapeutic compounds against primary human AML cells and to study the mechanisms of chemo-resistance.

Although AML xenotransplantation into immunodeficient murine models is a valuable tool for the expansion and study of some aspects of the biology of human AML, these models are still limited by their inability to address the interplay of leukemic blasts with different cells of the immune system and to dissect the cell autonomous from cell non-cell autonomous aspect of the disease as they tend to develop other spontaneous malignancies. To overcome these limitations, scientists took advantage of new advances in the fields of bioengineering and synthetic material development to create biological inserts or scaffolds [149]. The function of these scaffolds is to create humanized microenvironment in the mouse that is efficient in supporting implanted cells expansion and differentiation without altering their character and function. Successful primary AML cells engraftment was achieved using polyurethane scaffolds coated with freshly isolated human BM-derived mesenchymal stem cells (hMSC) in NOD-SCID mice [150]. The subcutaneously implanted scaffold remodelled the architecture of human BM niche (with de novo vascularization and osteoclast and adipocyte development) at the site of implantation and supported the initial expansion and spreading (BM, liver and kidney) of pre-implanted and retro-orbitally injected AML cells. In another study, hMSC coated ceramic scaffolds were able to support the engraftment of favourable non-engrafting AML samples when implanted subcutaneously in NSG mice [151]. The implanted insert supported cellular proliferation and maintained clonal heterogeneity and leukemic stem cells’ (LSC) self-renewal capacity in methylcellulose cultures. In a different approach, researchers also used freshly collected human BM biopsies from hip replacement patients and directly transplanted them subcutaneously into the flanks of NSG mice, using Matrigel as carrier [152]. The human BM tissue showed vascularization and rapid engraftment of intravenously injected MOLM-13 human AML cells.

In an attempt to study the heterogenous sub-clones detected in human patients [153] and follow the stage specific transformation of MDS to AML [154], scientists took advantage of induced pluripotent stem cells (iPSC) technique to establish AML- and MDS-iPSC respectively. Despite the challenges in deriving myeloid malignant IPS cells due to their inherent resistance to reprogramming and apoptotic priming in ex vivo culture, both studies showed that in a pluripotent state, AML-iPSC do not possess a transformed phenotype. However, these cells appear to retain their leukemic potential upon induced hematopoietic differentiation and are able to induce disease in NSG mice. Interestingly, when stimulated to differentiate towards non-hematopoietic lineage, AML-derived IPSC are able to form non-malignant cells from all three embryonic germ layers [153].

These novel approaches in leukemic remodelling using the classical PDX system have provided proof of concept solutions on how to overcome some of challenges associated with the system, such as the difference between the mouse and human BM microenvironment and AML samples the *intra* and *inter* heterogeneity.

## 6. AML Mouse Models Generated with Genome-Editing Techniques

The recent development of gene editing tools such as the clustered regularly interspaced short palindromic repeats (CRISPR) and the transcription activator-like effector nucleases (TALEN) offer novel tools to study the biology of AML by engineering disease-associated mutations in primary cells (Figure 1C,D). Pioneering work by the Ebert lab explored disease modelling by genome-editing of AML-associated mutations and inactivation of multiple tumour suppressor genes using a double lentiviral expression system [155]. Hereby, one vector delivered *Cas9* and a green fluorescent marker (eGFP), while the other carried the guide RNA (sgRNA) targeting the *Tet2*, *Runx1*, *Dnmt3a*, *Nf1*, *Ezh2* and *Smc3* genes in conjunction with another fluorescent marker (RFP-567). Viral transduction of lineage marker-depleted Sca1^+^; cKit^+^ (LSK) cells from C57B1/6 wild-type and *Flt3-ITD* knock-in mice with pooled sgRNA virus followed by transplantation into lethally irradiated recipients caused significant myeloid skewing of haematopoiesis and development of splenomegaly and leukaemia in some mice. Sequencing of genomic DNA from clonal leukemic cells revealed mutations in *Tet2*, *Dnmt3a*, *Runx1*, *Nf1* and *Ezh2* in single cells, thus indicating clonal outgrowth and transformation. This strategy was further refined to model mutations associated with CHIP [156]. Several CHIP and AML mutated genes (*Dnmt3a, Ezh2, Nf1, Runx1, Ascl1, Smc3* and/or Ep300) were edited simultaneously in murine HSPC, followed by transplantation into lethally irradiated recipients [156]. Genomic sequencing following long term observation and malignancy development showed single clonal expansion, especially from those harbouring *Dnmt3a* mutations. The mice showed a general increase in myeloid chimerism and clonal expansion reminiscence of CHIP. Some mice died of severe anaemia, while others progressively developed hematopoietic failure and AML. Genomic DNA sequencing detected deleterious mutations in all targeted genes except *Ep300*, leading to block in differentiation and activation of RAS-MAPK pathway [156]. A similar approach was used by another group to edit commonly mutated AML genes such as *TET2*, *ASXL1*, *DNMT3A*, *RUNX1*, *TP53*, *NF1*, *STAG2* and *SMC3* in human umbilical cord blood (UCB) and adult CD34^+^ cells, by introducing a pool of 11-targeted sgRNAs [157]. Consistent with patients’ data, in vitro generated colonies derived from single edited UBC carried bi-allelic loss-of-function (LOF) mutations in *TET2*, *DNMT3*, *EZH2*, *TP53* and *NF1* but only single allele mutations of *SMC3*, *ASXL1* and *RUNX1* were detected. To employ the multiplex genome editing in vivo, the researchers edited human adult CD34^+^ cells with the same pool of sgRNAs together with *FLT3-ITD* and mutated *NPM.* Transplantation of edited cells into immune-compromised NSG-S mice resulted in the development of CHIP and MDS. Genomic DNA screening of in vivo expanded clones showed mutagenic pattern similar to the in vitro experiments, with overrepresentation of clones carrying LOF mutation (mostly deletions leading to frame shift) in *TET2*, *DMNT3A* and *ASXL1*. Despite these promising observations, none of the mice developed AML. The authors suggested that differences between the human and murine BM microenvironment might impair in vivo leukemogenesis [157].

Another study used CRISPR/Cas9-based genome editing to model the recurrent *7q* deletion associated with MDS and AML [158]. The commonly deleted region contains the *mixed lineage leukaemia 3* (*MLL3*) gene but mutations and deletions of *MLL3* have been only detected on one allele, suggesting that *MLL3* functions as a haplo-insufficient tumour suppressor. To prove this hypothesis, researchers transduced tumour-prone (*p53^−/−^* with reduced expression of the tumour suppressor *Nf1*) HSPC with sgRNA targeting *Mll3* followed by transplantation into sub-lethally irradiated *C57Bl/6* mice. Compared to controls, targeting *Mll3* significantly accelerated leukaemia development. Subsequent gDNA sequencing of individual *Mll3* edited AML clones revealed both wild type and mutant alleles in the majority of samples. This suggested that leukemogenesis selects for partial but not complete *Mll3* inactivation, providing compelling evidence that *Mll3* is a haplo-insufficient tumour suppressor gene in AML [158].

Genome editing using TALEN effector nucleases was used to specifically generate reciprocal chromosomal translocations of the *MLL* and *AF9* genes (*MLL-AF9* and *AF9-MLL*) in primary human CD34^+^ UCB-derived HSC, to recapitulate MLL rearrangements in patients’ cells [159,160]. In the first study, edited HSPC showed heterogeneous response to the fusion whereby only some cells showed a clear proliferative advantage. The cells were not sufficiently transformed and could not be significantly expanded in culture and had a limited replating capacity in methylcellulose culture [159]. In contrast, in the second study, researchers were able to induce a leukemic phenotype by transplanting in vitro expanded monoclonal and immortalized cells into NSG mice. Notably, no secondary pathogenic mutations were found by targeted exome and RNA-sequencing, suggesting that this MLL fusion might be sufficient to initiate the disease [160]. Using engineered lentiviral vectors carrying Cas9 and two sgRNA sequences targeting the MLL and ENL locus researchers were able to generate the reciprocal t(11;19) translocation leading to expression of the MLL-ENL fusion in human CD34^+^ UCB cell [161]. Unfortunately, similar to the first study done with MLL-AF9 fusion, the cells did not display enhanced self-renewal capacity in vitro when cultured in methylcellulose media. However, when injected in sub-lethally irradiated NSG-S mice, they were able to produce leukaemia with monocytic features. Future work will show whether Crispr/Cas9 genome editing will be suitable for the generation of animal models carrying multiple functionally cooperating genetic lesions ultimately progressing into clinical AML.

## 7. Conclusions

None of the currently used mouse AML models faithfully recapitulate the complex biology, cell to microenvironment interactions and dynamic progression of AML. Nevertheless, they have been instrumental in deciphering the underlying pathology of the disease and advancing AML research. Historically, the chemical, irradiation and viral models set the field of AML modelling in mice and were used to develop many AML drugs. Transgenic mouse lines harbouring AML associated mutations have enabled researchers to directly link genetic aberrations to AML initiation and progression. The creation of immunocompromised mouse strain has allowed for the expansion and study of human primary AML cells and the discovery of a hierarchy led by leukemic stem cell. In the future, advancement in genome editing technologies and collaboration between multidisciplinary fields would lead to the generation of more humanized mouse strains, which will ultimately help scientists to accurately model the complex biology of AML in mice.

## Figures and Tables

**Table 1:** Transgenic mouse lines modelling AML.

Year	Transgene	Strategy	Promoter	Inducer	Cellular Target	Phenotype	Ref.
1996	*PML-RARA*	Conventional	*CD11b*		Myeloid lineage (BM, periphery)	Abnormal myelopoiesis. No APL	[52]
1997	*PML-RARA*	Conventional	*hCG*		Myeloid lineage (BM, periphery)	Myeloid cells expansion in BM and spleen. AML-like with 30% penetrance after long (> 100 days) latency	[51]
1997	*PML-RARA*	Conventional	*hMRP8*		Myeloid lineage (BM, periphery)	APL-like disease (median 174 days)	[53]
2000	*RUNX1-ETO*	Conditional	*Tet*	*tTA*	BM	Abnormal haematopoiesis. No AML	[60]
2001	*RUNX1-ETO*	Conventional	*hMRP8*		Myeloid (neutrophils & monocytes)	AML-Only upon new-born treatment with ENU	[61]
2006	*Cbfb-MYH11*	Conditional	*Cbfβ*	*Mx-iCre*	BM (LSK)	AML-Aberrant myeloid progenitors, blocked megakaryotic differentiation.	[62]
2008	*Mll-AF9*	Knock-in (*Mll1; Mllex8-AF9* cDNA)	*Mll*			AML-Higher *MLL-AF9* expression in HSCs than GMPs.	[63]
2014	*MLL-ENL*	Conditional	*TRE (Col1a)*	*rtTA*	LT-HS, pMeg/E, HSC, MPP, GMLP, CLP	AML- no leukaemia from HSC	[64]
2016	*MLL-AF9*	Conditional	*TRE (Hprt)*	*rtTA*	LT-HSC, ST-HSC, CMP, GMP	AML-dependent on DOX dose and cellular origin	[65]
2018	*MLL-ENL*	Conditional	*TRE (Hprt)*	*rtTA*	LT-HSC, LMPP, CMP	AML-MLL-dependent on DOX dose and cellular target	[66]

Conventional (DNA injection into Oocytes), Knock-in (homologous DNA recombination in ES cells), Conditional (regulated expression), LSK (lineage marker negative, Sca1^+^, cKit^+^), MPP (multipotent progenitors), GMLP (granulocyte-macrophage-lymphoid progenitors), CLP (common lymphoid progenitor), ST-HSC (short term hematopoietic stem cells), DOX (doxycycline).

**Table 2:** Compound transgenic mouse AML models.

Year	Co-Op Mutations	Activity	Promoter	Inducer	Cellular Target	Phenotype	Ref.
2007	*NRAS12D + BCL2*	Const.	*hMPP8*		Myeloid lineage (BM, periphery)	MDS/AML	[102]
		Cond.	*Tet*	*rtTA*			
2012	*MLL-PTD + FLT3-ITD*	Const.	*Mll + Flt3*		*Mll* and *Flt3* expressing cells	AML with 100% penetrance	[97]
2012	*NUP98-HOXD13 + FLT3-ITD*	Conv. (*FLT3-ITD*)	*Flt3*		Hematopoietic lineage cells (FL, BM)	AML with 100% penetrance	[98]
		Conv. (*NUP98-HOXD12*)	*Vav*				
2012	*KRAS-G12D + PML-RARA*	Cond. (*Kras-G12D*)		*Mx-iCre*	Myeloid lineage (BM, periphery)	APL-like Disease with 69% penetrance, remaining mice developed MDS	[104]
		Const. (*PML-RARA*)	*hCG*				
2013	*NPM1c + FLT3-ITD*	Cond. (*NPM1c*)	*Mx1*	*Mx-iCre*	Hematopoietic lineage cells (BM)	AML after short latency (median 49 days)	[96]
		Const. (*Flt3-ITD*)					
2014	*NRAS-G12D + CBFβ-SMMHC*	Cond.	*Mx1*	*Mx-iCre*	Hematopoietic lineage cells (BM)	AML after short latency (median 13.7 weeks) and full penetrance	[103]
2017	*NPM1c + NRAS-G12D*	Cond.	*Mx1*	*Mx.iCre*	Hematopoietic lineage cells (BM)	AML with 95% penetrance, some mice develop MPN	[46]
	*NPM1c + FLT3-ITD*					AML with 100% penetrance	
2018	*WT1-R394W + FLT3-ITD*	Const.	*Wt1* and *Flt3*		Wt1 and Flt3 expressing cells	MPN-like disease or T-ALL after short latency-AML associated with LOH of *Flt3*	[99]

Const. (constitutive expression), Cond. (regulated expression), Conv. (conventional), FL (fetal liver), MPN (myeloproliferative neoplasms), T-ALL (T-cell acute lymphoblastic leukemia).

**Table 3:** AML mouse models based on viral transduction and transplantation.

Year	Transgene	Viral Vector	Cellular Target	Phenotype	Ref.
1990	*BCR-ABL*	*pMSCV-pgk-neo*	Total BM	Myeloproliferative malignancy, ALL and CML-like	[111]
1997	*MLL-ENL*	*pMSCV-IRES-GFP*	*Thy-1^lo^Sca-1^+Hi^-2K^hi^, 5-FU treated BM*	Self-renewal in vitro & AML in vivo	[117]
2002	*RUNX1-ETO*	*pMSCV-IRES-GFP*	*HSC c-Kit* *^+^* *Sca-1* *^−^* *Lin* *^−^*	Myeloid developmental abnormality but no AML	[118]
2003	*MLL-GAS7*	*pMSCV-pgk-neo*	*HSPC*	Mixed lineage leukaemia phenotype	[120]
2004	*MOZ-TIF2, BCR-ABL*	*pMSCV-IRES-GFP*	*CMP, GMP*	MOZ-TIF2 but not BCR-ABL resulted in transplantable AML in vivo	[114]
2006	*MLL-AF9*	*pMSCV-IRES-GFP*	*GMP*	Transplantation of transduced cells propagated in MC resulted in AML in vivo	[115]
2011	*MN1*	*pMSCV-pgk-neo*	*CMP, GMP*	CMP are susceptible for MN1 transformation, GMP required co-expression of MEIS1 for AML induction	[121]
2012	*MLL-AF9*	*pMSCV-pgk-puro*	*Evi1^+/−^* MLL-AF9 transduced cells	Knockdown of *Evi1* delayed leukaemia induction in vivo	[116]

FU (Fluorouracil), MC (methylcellulose).
